# Influence of Drug Resistance Mutations on the Activity of HIV-1 Subtypes A and B Integrases: a Comparative Study

**Published:** 2015

**Authors:** O. A. Shadrina, T. S. Zatsepin, Yu. Yu. Agapkina, M. G. Isaguliants, M. B. Gottikh

**Affiliations:** Faculty of Bioengineering and Bioinformatics, Lomonosov Moscow State University, Leninskie gory, Moscow, 119991, Russia; Belozersky Institute of Physical-Chemical Biology, Lomonosov Moscow State University, Leninskie gory, Moscow, Russia; 119991; Chemistry Department, Lomonosov Moscow State University, Leninskie gory, Moscow, 119991, Russia; Ivanovsky Institute of Virology, Gamaleya Str., Moscow, 123098, Russia; Department of Microbiology, Tumor and Cell Biology, Karolinska Institutet, Stockholm, 17177, Sweden

**Keywords:** integrase, HIV-1 subtype A, strain FSU-A, strand transfer inhibitor, drug resistance mutations

## Abstract

Integration of human immunodeficiency virus (HIV-1) DNA into the genome of an infected cell is one of the key steps in the viral replication cycle. The viral enzyme integrase (IN), which catalyzes the integration, is an attractive target for the development of new antiviral drugs. However, the HIV-1 therapy often results in the IN gene mutations inducing viral resistance to integration inhibitors. To assess the impact of drug resistance mutations on the activity of IN of HIV-1 subtype A strain FSU-A, which is dominant in Russia, variants of the consensus IN of this subtype containing the primary resistance mutations G118R and Q148K and secondary compensatory substitutions E138K and G140S were prepared and characterized. Comparative study of these enzymes with the corresponding mutants of IN of HIV-1 subtype B strains HXB-2 was performed. The mutation Q148K almost equally reduced the activity of integrases of both subtypes. Its negative effect was partially compensated by the secondary mutations E138K and G140S. Primary substitution G118R had different influence on the activity of proteins of the subtypes A and B, and the compensatory effect of the secondary substitution E138K also depended on the viral subtype. Comparison of the mutants resistance to the known strand transfer inhibitors raltegravir and elvitegravir, and a new inhibitor XZ-259 (a dihydro-1H-isoindol derivative), showed that integrases of both subtypes with the Q148K mutation were insensitive to raltegravir and elvitegravir but were effectively inhibited by XZ-259. The substitution G118R slightly reduced the efficiency of IN inhibition by raltegravir and elvitegravir and caused no resistance to XZ_259.

## INTRODUCTION

Integrase (IN) is one of the key enzymes of human immunodeficiency virus type 1 (HIV-1) required for its replication. IN catalyzes the insertion of a DNA copy of the viral genomic RNA into the host DNA in two consecutive reactions. The first reaction is the 3’-processing, consisting in the GpT dinucleotide cleavage from both 3’-ends of the viral DNA. The second reaction is the strand transfer, in which the viral DNA is inserted into the host cell’s DNA.

Since IN homologues within human cells have not been described, IN is an attractive target for developing new antiviral drugs. Three strand transfer inhibitors are currently used as components of highly active antiretroviral therapy: raltegravir (RAL), elvitegravir (EVG), and dolutegravir (DTG). However, strand transfer inhibitors cause drug resistance mutations in the IN gene both in patients and in a HIV-infected cell culture [1]. The virus rapidly develops resistance, including cross-resistance, to the first generation of strand transfer inhibitors – RAL and EVG. One of the common reasons for the high resistance to both inhibitors is a primary mutation at the Q148 residue [2-6]. In most cases, this mutation occurs in combination with secondary mutations, most frequently G140S/A and E138K/A [2-7]. The results of *in vitro* and *in vivo *studies have demonstrated that secondary mutations partially restore the viral replication ability reduced by primary substitutions and may also increase drug resistance [7-11].

DTG is a second-generation drug active against most RAL- and EVG-resistant virus strains [9, 12, 13]. However, investigation of the DTG effect on HIV-1 isolates from patients insensitive to RAL and EVG showed that Q148H/K/R substitutions in the integrase structure lead to some resistance to DTG. Secondary and tertiary mutations (G140A/C/S, L74I and E138A/K/T) further enhance the resistance [14, 15]. Variants containing a number of amino acid substitutions in IN (H51Y, L101I, G118R, T124A, S153F/Y, R263K) were found during selection of HIV-1 strains resistant to DTG in a lymphocytes culture [13, 16]. However, only two substitutions, G118R and R263K, proved to be responsible for the virus resistance to DTG [15, 17].

HIV-1 is represented by different subtypes and recombinant strains, and among them subtype B is prevalent in the United States, Australia, Japan, and Western Europe. Mutations Q148H/R/K lead to RAL- and EVG-resistance in different HIV-1 subtypes. Mutations associated with DTG-resistance are more specific. Thus, *in vitro *selection of DTG-resistant strains of HIV-1 subtypes B, C, and A/G demonstrated that only the R263K substitution was common to all subtypes; the G118R substitution was found only in the subtypes A/G and C [16]. In subtype C, this mutation was found also by *in vitro*-selection with the second-generation strand transfer inhibitor MK-2048 [18]. The same study demonstrated that the E138K mutation was a secondary compensatory substitution for G118R. The fact that the G118R mutation is associated with the lack of sensitivity to RAL in patients infected with the CRF02_A/G strain has recently been demonstrated [19]. It is important to note that this virus isolate, containing the G118R substitution in the IN gene, was resistant not only to RAL, but also to EVG and DTG [15]. All these data suggest that the G118R substitution is most characteristic for non-B subtypes of HIV-1 and that the presence of this substitution can lead to patient insensitivity to all IN inhibitors approved for therapeutic use.

HIV subtype A (FSU-A) dominates within the territory of the former Soviet Union, and IN of this viral subtype has not been fully characterized [20]. In particular, information on resistance mutations caused by IN inhibitors in HIV-1 strain FSU-A is limited. To assess the impact of drug resistance mutations on the enzymatic properties of IN of HIV-1 subtype A, we prepared a consensus IN of the FSU-A strain, where RAL- and EVG-resistance mutations were introduced by site-directed mutagenesis [21, 22]. The consensus IN sequence of HIV-1 strain FSU-A (INA) differs from the sequence of the best studied IN of HIV-1 subtype B (HXB-2) by substitutions of 16 amino acid residues, nine of which are located in the catalytic domain. We characterized the catalytic activity of INA and its variants containing two major combinations of RALand EVG-resistance mutations: E92Q, V151I, N155H, G163R, L74M (mutant 1), and Q148K, E138K, G140S (mutant 2) [22]. The consensus enzyme was significantly more active than IN of subtype B (INB) in 3’-processing and strand transfer reactions. The introduction of these mutations significantly increased INA resistance to RAL and EVG but dramatically reduced its catalytic activity in both reactions [22].

In this study we continued the investigation of the role of drug resistance mutations and meticulously compared the effect of the primary mutation Q148K and the secondary mutations E138K and G140S on the activity of IN_A_ and IN_B_. We also described the activity of the IN_A_ mutants containing the primary G118R substitution and compensatory E138K substitution for the first time. The Q148K mutation dramatically decreased the activity of enzymes of both viral subtypes in both reactions: 3’-processing and strand transfer. This decrease was partially restored by the secondary mutations E138K and G140S. The G118R substitution reduced the efficiency of 3’-processing for both integrases by 5 times, but it differently affected the enzymes of different strains in the strand transfer reaction: IN_A_ activity decreased more significantly than IN_B_ activity. Moreover, the secondary substitution E138K had a compensatory effect on IN_B_ only. We also compared the resistance of all the mutants to RAL, EVG, and the new strand transfer inhibitor XZ-259 [23]. XZ- 259 effectively inhibited the RAL- and EVG-resistant IN forms containing substitution Q148K. Substitution G118R slightly reduced the efficiency of IN inhibition by RAL and EVG, this effect was more pronounced in the case of IN_B_, and did not affect the sensitivity of INs to XZ-259. ;

## MATERIALS AND METHODS

**Enzymes**

Plasmid vector pET-15b (Novagen, USA) was used for expression of recombinant INs (wt and mutants) of both HIV-1 subtypes with N-terminal His6-tag . Protein samples were isolated from cells of the Rosetta (DE3) *Escherichia coli *producer strain and purified without adding a detergent as per [24]. Genetic constructs encoding IN mutant forms were obtained by site-directed mutagenesis of a plasmid encoding corresponding wild-type IN using a QuikChange II Site-Directed Mutagenesis kit (Agilent Technologies, USA). All procedures were performed in accordance with the manufacturer’s instructions. Preparations were analyzed by electrophoresis in 12% SDS-PAGE according to Laemmli, followed by staining with SimplyBlueTM SafeStain (Invitrogen, USA) according to the manufacturer’s instruction. The purity of the IN preparations was not lower than 90%.

**Oligodeoxyribonucleotides**

All oligodeoxyribonucleotides were synthesized using the phosphoramidite method on an ABI 3400 DNA synthesizer (Applied Biosystems, USA) in accordance with the standard operating procedures using commercially available reagents (Glen Research, USA). ;

The radioactive ^32^P-label was introduced at the 5’- end of the oligonucleotides. To achieve this, 10 pmol of the oligonucleotide was incubated with T4-polynucleotide kinase (Fermentas, Lithuania) and 50 μCi (16 pmol) [γ-^32^P]ATP (3000 Ci/mmol), in 10 μl of a buffer containing 50 mM Tris-HCl, pH 7.5, 10 mM MgCl_2_, 5 mM DTT, 0.1 mM spermidine, 0.1 mM EDTA, for 1 h at 37°C. Then, the kinase was inactivated by adding 2 μl of 250 mM aqueous EDTA and heating to 65°C for 10 min. An equimolar amount of the complementary oligonucleotide was added, and a duplex was formed by heating the oligonucleotide mixture to 95°C followed by slow cooling to room temperature. The duplex was purified from the excess [γ-^32^P]ATP and salts on a MicroSpin G-25 column (Amersham Biosciences, USA) according to the manufacturer’s instructions.

**HIV-1 IN catalytic activity assays**

Duplex U5B/U5A consisting of 21-mer oligonucleotides U5B (5’-GTGTGGAAAATCTCTAGCAGT-3’) and U5A (5’-ACTGCTAGAGATTTTCACAC-3’) and mimicking the end of the HIV-1 U5 LTR was used as a substrate for the 3′-processing. For this reaction, 3 nM duplex U5B/U5A (with 32P-labeled U5B-chain) was incubated with 100 nM IN in 20 μl of a buffer (20 mM HEPES, pH 7.2, 7.5 mM MgCl2, 1 mM DTT) at 37°C. The incubation time varied from 1 to 2,000 min. The reaction was stopped by adding 80 μl of the buffer containing 7 mM EDTA, 0.4 M sodium acetate, 10 mM Tris-HCl, pH 8, and 0.1 g/l glycogen (stop solution). The IN protein was extracted with phenol: chloroform: iso-amyl alcohol = 25: 24: 1, the DNA duplex was precipitated with ethanol (250 μl). The reaction products were separated by electrophoresis in a 20% polyacrylamide/7 M urea gel in the TBE buffer. Autoradiographic data analysis was performed using a GE Typhoon FLA 9500 scanner; densitometry was performed using the ImageQuant 5.0 software. The efficiency of 3’-processing was determined as the intensity ratio of the bands corresponding to the U5B substrate and the reaction product U5B-2 truncated by two residues using the ImageQuantTM 5.0 software. The statistical analysis was performed using the Gnuplot version 4.6.

For the homologous strand transfer reaction, the U5B-2/U5A duplex was used as both a DNA substrate and a target. The reaction was carried out in the buffer used for 3’-processing with the 10 nM U5B-2/U5A duplex (with ^32^P-labeled U5B-2 chain) and 100 nM IN at 37°C; aliquots were taken after 2, 4, and 6 h.

For the heterologous strand transfer reaction, U5B-2/U5A and 36-bp duplex DNA (5’-ACAAAATTCCATGACAATTGTGGTGGAATGCCACTA- 3’, 5’TAGTGGCATTCCACCACAATTGTCATGGAATTTTGT- 3’) were used as a DNA substrate and a target respectively. The U5B-2/U5A substrate (2 nM, ^32^P-labeled U5B-2chain) was first incubated in the buffer for 3’-processing with 100 nM IN at 25°C for 30 min; the target DNA (8 nM) was then added, and the mixture was incubated for 2 h at 37°C. The reaction products were isolated and analyzed as described above.

**Inhibition of the strand transfer reaction**

The resistance of INs to inhibitors, RAL, EVG (Santa Cruz Biotechnology Inc., USA) and XZ-259 (kindly provided by Dr. Xue Zhi Zhao from NIH, USA), was investigated in the homologous strand transfer reaction carried out as described above for 2 h in the presence of increasing inhibitor concentrations. Using the results of three independent determinations, IC50 values were determined for each inhibitor. Data for the reaction efficiency were approximated by the exponential decay function; the concentration value corresponding to 50% of inhibition was calculated.

## RESULTS AND DISCUSSION

Fourteen mutant proteins (seven for each IN: Q148K, G140S, E138K, G118R, Q148K/E138K, Q148K/G140S, and G118R/E138K) were prepared by site-directed mutagenesis for the comparative analysis of the effect of drug resistance mutations on the catalytic activity of INs of FSU-A (IN_A_) and HXB-2 (IN_B_) strains. Enzymatic activities were determined in 3’-processing and strand transfer reactions using synthetic DNA duplexes corresponding to the end of the U5 region of the viral cDNA long terminal repeat .

**Mutations influence on the catalytic activity of IN_A_ and IN_B_ in the 3’-processing reaction**

We used a 21-mer DNA duplex U5B/U5A mimicking the U5 region of HIV-1 DNA (U5-substrate) and the conditions (enzyme and DNA concentrations, buffer composition) described earlier for the analysis of catalytic activities of IN_A_ and IN_B_ [22] in the 3′-processing reaction.

**Fig. 1:**
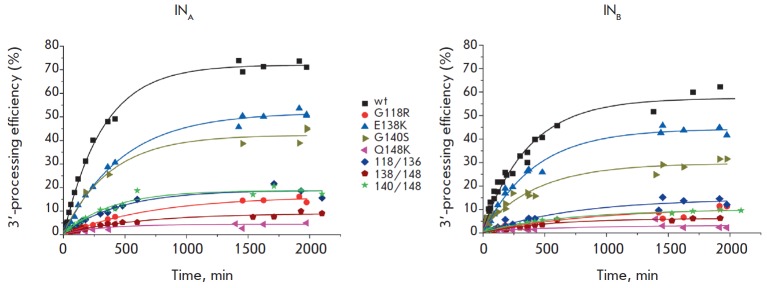
The kinetics of 3’-processing product accumulation catalyzed by consensus IN of HIV-1 subtype A strain FSU-A (IN_A_) and IN of HIV-1 subtype B strain HXB-2 (IN_B_) and their mutants. The reaction was carried out at 37°C using 100 nM IN and 3 nM U5 substrate. The average values of at least three independent measurements with a standard error of less than 12% are shown

We evaluated the dependence of the 3’-processing efficiency on time and plotted kinetic curves for product accumulation (*Fig. 1*). The initial rates of the 3’-processing reaction (*V0*) were calculated from the linear part of the curve (first 60 min) (*Table 1*).

**Table 1:** Initial rates and efficiencies of 3’-processing catalyzed by IN_A_ and IN_B_ and their mutants

Mutation	V_0_, pM/min*		Relative reaction efficiency, %**	
	IN_A_	IN_B_	IN_A_	IN_B_
Wild type	10.1 ± 0.29	6.4 ± 0.19	100	100
G118R	0.98 ± 0.074	0.79 ± 0.15	21	20
E138K	4.8 ± 0.24	4.6 ± 0.9	69	76
G118R/E138K	2.6 ± 0.37	1.4 ± 0.18	24	24
G140S	4.3 ± 0.21	4.8 ± 0.75	58	51
Q148K	0.90 ± 0.16	0.65 ± 0.35	6	13
E138K/Q148K	1.2 ± 0.31	0.7 ± 0.61	13	11
G140S/Q148K	2.62 ± 0.11	1.3 ± 0.23	25	15

*Mean values of at least three independent experiments with standard deviations are shown.

**Relative reaction efficiency after 1,500 min of incubation is shown; efficiency of the reaction catalyzed by wt IN is 100%.

As we demonstrated earlier [22], INA was more active than INB in the 3’-processing reaction. All IN_A_ mutants were also characterized by a higher efficiency of product accumulation than the corresponding IN_B_ mutants (*Fig. 1*). However, the initial reaction rates for mutant forms of both INs were not significantly different (*Table 1*).

All mutations introduced into INs of both subtypes reduced both the 3’-processing rate and the efficiency of product accumulation (*Fig. 1*, *Table 1*). The most significant decrease was detected for proteins with the Q148K substitution; this finding is in good agreement with the previous results for IN_B_ [25].

As we expected based on published data [7-11, 13], the negative effect of the primary mutation Q148K was partially recompensed by the G140S substitution (*Fig. 1*, *Table 1*). The compensatory effect of G140S was stronger for IN_A_: the difference in the 3’-processing efficiency and initial rate for mutants IN_A_^G140S/Q148K^ and IN_A_^Q148K^ was more pronounced than that for the corresponding pair of subtype B (*Fig. 1,* *Table 1*). However, it should be noted that the compensatory effect of G140S on the Q148K mutation observed for IN_A_^Q148K^ and IN_B_^Q148K^ was not as significant as on the Q148H substitution in IN_B_ [8]. This may be explained by the stronger negative impact of the Q148K mutation on the IN activity. The difference in the activities of IN with the primary mutations Q148K and Q148N correlated with the differences in the integration capacity of viruses carrying these mutations [7, 10, 11].

A compensatory effect of E138K on the catalytic activity of both INs with the primary Q148K substitution was also detected (*Fig. 1*, *Table 1*). However, both double mutants INA E138K/Q148K and IN_B_^E138K/Q148K^ were less active than the double mutants carrying the G140S/Q148 substitutions. This finding is consistent with a decrease in the replication and integration activity of HIV-1 subtype B mutants in the series: Q148K < Q148K/E138K < Q148K/G140S [7]. Interestingly, activity of INA with triple mutation E138K/G140S/Q148K was slightly higher than that of the enzymes with two substitutions: 1,500 min after initiation of the reaction, the 3’-processing efficiency for the triple mutant was about 30% of that for the wt IN_A_ [22], while for the most active double mutant IN_A_^G140S/Q148K^ it was not higher than 20% (Table 1). Thus, the compensatory effect of the combination of two mutations, E138K and G140S, was slightly higher than that of the individual secondary substitution, G140S or E138K. A similar observation was made earlier for HIV-1 subtype B: the addition of the E138K mutation to the Q148K/G140S substitutions improved viral replication while not affecting viral sensitivity to strand transfer inhibitors [11].

Finally, we found that the G118R substitution strongly decreased the activities of both INA and INB (*Fig. 1*, *Table 1*). This result contradicts the data reported in [17], which demonstrated that the efficiency of 3’-processing catalyzed by recombinant IN_B_ with the G118R substitution was slightly reduced, whereas the double mutants G118R/E138K and G118R/H51Y were somewhat more active than the wt enzyme. Under our conditions, the introduction of the secondary E138K substitution also led to increased activities of both the IN_A_^G118R^ and IN_B_^G118R^ mutants; however, the activities of all enzymes with the G118R substitution were significantly lower than those of wt IN_A_ and IN_B_ (*Fig. 1*, *Table 1*). This contradiction can be explained by the different 3’-processing conditions; in particular, by the length of the DNA substrate: we used a standard 21-mer DNA duplex, while a 32-mer substrate was used in [17].

**Effect of mutations on the catalytic activities of IN_A_ and IN_B_ in the strand transfer reaction**

**Fig. 2:**
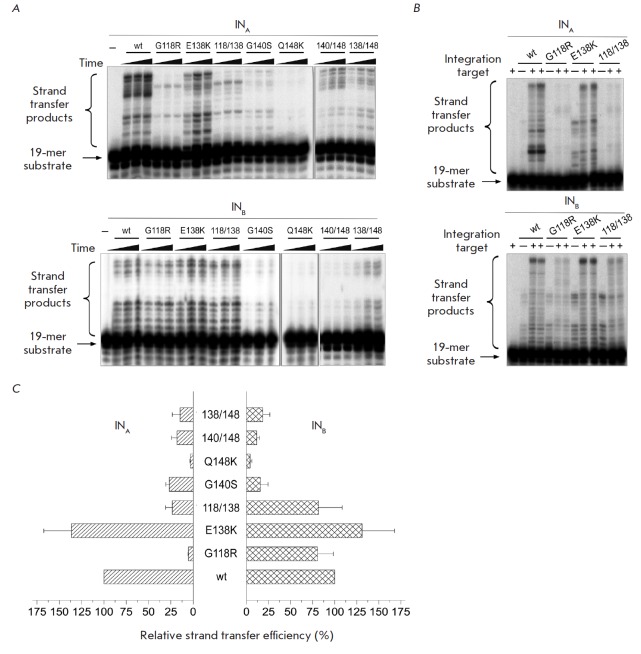
The catalytic activity of the mutant INs of HIV-1 subtypes A and B in the strand transfer reaction. All products were resolved by electrophoresis in 20% PAAG under denaturing conditions. **A. **Reaction of homologous strand transfer was performed at 37Ѓ‹C for 2, 4, 6 h using 100 nM IN and 10 nM substrate U5B-2/U5A. **B. **Reaction of heterologous strand transfer was performed using 100 nM IN, 2 nM substrate U5B-2/U5A (pre-incubated for 30 min at 25Ѓ‹C) and 8 nM 36-mer DNA target for 2 h at 37Ѓ‹C. **C. **Relative efficiency of homologous strand transfer catalyzed by the mutant INs: the reaction efficiency for wt IN_A_ and IN_B_ is considered to be 100%. The average values of at least three independent measurements with the standard error are shown

We also investigated the mutations effect on the second reaction catalyzed by IN, which is the strand transfer. In *in vitro *reaction, the 3’-processed DNA substrate may be inserted by IN into itself (homologous strand transfer) or into any random DNA duplex or plasmid (heterologous strand transfer). The U5B-2/U5A duplex was used as a DNA substrate. A synthetic 36-mer oligonucleotide duplex was used as a target for heterologous strand transfer. Since the sites of the substrate insertion do not depend on the DNA target sequence, reaction products with different lengths were detected (*Fig. 2*).

As we established earlier [22], IN_A_ activity was slightly higher than that of IN_B_ in the strand transfer reaction (*Fig. 2*). A difference in the profiles of the integration products for the homologous (*Fig. 2A*) and heterologous strand transfer (*Fig. 2B*) catalyzed by IN_A_ and IN_B_ can be observed.

INs of both subtypes carrying the Q148K substitution were the least active in the strand transfer reaction, identically to 3’-processing. For these mutants, the efficiency of homologous strand transfer was reduced to approximately 5% of that of the wt enzymes. Surprisingly, the G140S substitution significantly decreased the reaction efficiency, too (*Fig. 2 A,C*). This effect was observed for the enzymes of both subtypes, IN_A_ G140 and INB G140, though no data on a G140S negative effect on the activity of recombinant IN have been published, and only a slight decrease in the integration and replication capabilities was demonstrated for HIV-1 subtype B with this substitution [7, 8]. Despite the negative effect of the G140S substitution, its combination with the Q148K mutation increased the reaction efficiency and the double mutants IN_A_^G140S/Q148K^ and IN_B_^G140S/Q148K^ were more active than IN_A_^Q148K^ and IN_B_^Q148K^ (*Fig. 2C*). Some compensatory effect was also produced by the E138K mutation. Moreover, the compensatory effect of G140S was somewhat stronger for IN_A_, while the compensatory effect of E138K was stronger for for IN_B_ (*Fig. 2C*). It is interesting to note that a single E138K substitution significantly increased the reaction efficiency for INs of both subtypes (*Fig. 2C*). In general, the primary mutation Q148K and its compensatory substitutions G140S and E138K equally affected the activities of IN_A_ and IN_B_ during 3’-processing and strand transfer reactions. Thus, the differences in the primary structure of IN_A_ and IN_B_ did not affect the enzymatic properties of this group of mutants *in vitro*.

It is important that another group of mutations, G118R and G118R/E138K, exhibited a different effect on the activity of INs of different subtypes in strand transfer reactions. IN_A_ was more sensitive to the G118 substitution than INB: the reaction efficiency was strongly reduced for the IN_A_^G118R^ enzyme, while it was not changed significantly for IN_B_^G118R^ (*Fig. 2 A,C*). It should also be noted that in the case of IN_A_, the G118R mutation resulted in a changed integration profile, and only two predominant products were detected for IN_A_^G118R^ instead of the large set of products found for wt IN_A_ (*Fig. 2A*). The addition of the compensatory mutation E138K had virtually no effect on the activity of the IN_B_^G118R^ mutant, while the double mutant IN_A_^G118R/E138K^ was more active than IN_A_^G118R^ carrying a single substitution. However, the efficiency of the homologous strand transfer catalyzed by IN_A_^G118R/E138K^ was only 23% of the reaction catalyzed by the wt IN_A_ (*Fig. 2C*).

It was shown previously that G118R substitution in IN_B_ significantly (over 90%) reduces its activity in the heterologous strand transfer reaction [17]. The double mutation G118R/E138K resulted in partial recovery of the activity, but it failed to achieve even 50% of the wt IN activity [17]. Similar effects were observed for HIV-1 subtype B containing these mutations: G118R substitution caused a significant decrease in the viral replication and integration, and the addition of the E138K mutation led to their partial recovery [18]. Our study of the G118R effect on the ability of IN_A_ and IN_B_ to catalyze the heterologous strand transfer showed that, identically to the homologous strand transfer, the effect of this substitution on the enzymes of different HIV-1 subtypes is different (*Fig. 2B*). The G118R mutation decreased IN_B_ activity by approximately 50%, while the corresponding IN_A_^G118R^ mutant was virtually inactive. The secondary substitution E138K had a compensatory effect only on INB: the activity of the IN_B_^G118R/E138K^ double mutant was somewhat higher than that of the IN_B_^G118R^ mutant (*Fig. 2B*). These results are consistent with data [17], and the difference in the activities of IN_B_ mutant forms (in our work and [17]) can be explained by differences in the reaction conditions. As for subtype A IN mutants, IN_A_^G118R^ and IN_A_^G118R/E138K^, they demonstrated equally low activities, although the substitution E138K alone resulted in increased efficiency of heterologous strand transfer catalyzed by INs of both subtypes (*Fig. 2B*).

The reduced integration activity of the subtype B mutant IN_B_^G118R^ had been explained by the reduced ability of the complex of this mutant with its DNA substrate to bind the DNA target [17]. As a result of natural polymorphism, INB contains Ser at position 119 and IN_A_ contains Pro [21]. It should be noted that Ser119 is likewise present in drug-resistant strains of HIV-1 subtype C, which most often contain the G118R mutation [16, 18]. The proline residue increases the rigidity of the IN spatial structure in the vicinity of the active site (Asp116 is a component of the catalytic triad). The Pro119 and G118R mutations obviously affect the ability of IN_A_ to interact with the DNA target to a higher extent than a combination of Ser119 and G118R. As a result, IN_A_ containing a G118R substitution is significantly less active in the strand transfer reaction than the corresponding IN_B_ mutant.

**The effects of mutations on the sensitivity of IN_A_ and IN_B_ to strand transfer inhibitors**

We have studied the influence of the selected drug resistance mutations on the IN sensitivity to three strand transfer inhibitors: RAL, EVG, and the new inhibitor XZ-259, a dihydro-1H-isoindole derivative, with biochemical and antiviral activities comparable to RAL [23]. We determined the concentration of the inhibitor required to reduce IN activity by 50% (IC_50_) in the homologous strand transfer reaction (Table 2; increased IC_50_ shows a decreased sensitivity of the enzyme to the inhibitor).

Our results demonstrate that IC_50_ values for RAL and EVG were comparable for INs of both subtypes, but the average IN_A_ sensitivity to both inhibitors was somewhat higher; this finding correlates with the data obtained previously [22]. IN_A_ sensitivity to the new inhibitor XZ-259 was also slightly higher than that of IN_B_; the IC_50_ value for INB (65 nM, Table 2) is in good agreement with [23] (77 nM).

**Table 2:** Inhibition of the activity of IN_B_, IN_A_ and their mutants in the reaction of homologous strand transfer by RAL, EVG, and XZ-259

Mutation	Inhibitory activity, IC_50_ * (nM), and ratio of IC_50_ for mutants over wt (FC)											
	IN_A_						IN_B_					
	RAL		EVG		XZ-259		RAL		EVG		XZ-259	
	IC_50_	FC	IC_50_	FC	IC_50_	FC	IC_50_	FC	IC_50_	FC	IC_50_	FC
Wild type	5 ± 2	1	17 ± 5	1	40 ± 15	1	7 ± 3	1	25 ± 10	1	65 ± 10	1
G118R	12 ± 5	2.4	45 ± 10	2.6	40 ± 10	1	30 ± 10	4.3	90 ± 30	3.6	80 ± 20	1.2
E138K	7 ± 3	1.4	35 ± 5	2	50 ± 15	1.25	7 ± 5	1	20 ± 8	0.8	70 ± 10	1
G118R/E138K	7 ± 3	1.4	40 ± 10	2.4	30 ± 10	0.75	25 ± 8	3.6	50 ± 15	2	80 ± 15	1.2
G140S	15 ± 5	3	300 ± 50	18	150 ± 50	3.8	35 ± 15	5	200 ± 80	8	150 ± 50	2.3
Q148K	400 ± 100	80	700 ± 80	41	350 ± 100	8.8	1100 ± 250	157	1000 ± 200	40	600 ± 100	9.2
E138K/Q148K	350 ± 80	70	650 ± 100	38	200 ± 50	5	500 ± 150	71	600 ± 150	24	500 ± 200	7.7
G140S/Q148K	400 ± 150	80	450 ± 150	26	600 ± 150	15	1000 ± 200	200	850 ± 200	34	850 ± 100	13

*Values are the average results of at least three independent determinations ± standard deviation.

It is convenient to use the FC values indicating by how much the IC_50_ value for a particular mutant has changed compared to the wild-type (i.e., a higher resistance of mutants to inhibitors in comparison with the wt enzyme) to analyze IN sensitivity to inhibitors. FC analysis of the protein family containing the primary substitution Q148K (INQ148K, IN^E138K/Q148K^ and IN^G140S/ Q148K^) showed that the resistance of the mutant INs of both subtypes to EVG increased in a similar manner (Table 2). RAL inhibited IN_A_ carrying the Q148K and G140S/Q148K substitutions twice more effectively than the corresponding IN_B_ variants. A compensatory E138K mutation decreased the resistance of IN_B_^Q148K^ to RAL and EVG almost twofold, without a significant effect on the resistance of the IN_A_^Q148K^ mutant. It should also be noted that the sensitivity of both Q148K mutants to XZ-259 was significantly higher than the sensitivity to EVG and especially to RAL; these results were in agreement with the results obtained earlier for IN_B_ [23]. It is interesting to note that the secondary E138K substitution increased the sensitivity of the IN_A_^Q148K^ and IN_B_^Q148K^ mutants to XZ-259, while G140S reduced their sensitivity (Table 2).

The FC analysis of the protein family with G118R and G118R/E138K substitutions showed a slight decrease in the sensitivity of both subtypes INs to RAL and EVG (Table 2). A single G118R mutation reduced the IN_B_ sensitivity more significantly (Table 2). Interestingly, the compensatory E138K substitution reduced the emerging resistance (Table 2). It is also important to note that resistance to XZ-259 did not occur. In general, our results correlate well with previously published data. Thus, the HIV-1 subtype CRF02_A/G isolate carrying a G118R substitution in the IN gene was resistant (FC>100) to all IN inhibitors approved for therapeutic use: RAL, EVG, and DTG [15]. Meanwhile, the HIV-1 subtype B (clone pNL4-3) carrying this mutation showed negligible resistance to these inhibitors (FC = 3.1 for EVG, 8.2 for RAL and 10 for DTG) [15]. Thus, our study confirms the heterogenic effect of the primary G118R mutation on the drug resistance of different HIV-1 subtypes.

## CONCLUSIONS

We have carried out the first systematic study of the enzymatic properties of consensus IN of HIV-1 subtype A strain FSU-A, which is dominant in the territory of the former Soviet Union, containing mutations G118R and Q148K causing HIV-1 resistance to strand transfer inhibitors. We have demonstrated that the sensitivity of IN_A_ to the inhibitors approved for therapeutic use, RAL and EVG, as well as to the novel inhibitor XZ-259, is somewhat higher than the sensitivity of IN_B_. The primary mutation Q148K associated with resistance to RAL and EVG caused a sharp decrease in IN_A_ activity, which is partially restored by the secondary mutations E138K and G140S. A similar dependence was observed for IN_B_. At the same time, the primary mutation G118R reduced the integration activity of IN_A_ much more significantly than the activity of IN_B_. This may be due to the IN natural polymorphism , and in particular to the presence of Pro119 in IN_A_ instead of Ser119 in IN_B_. We can assume that the Ser119Pro substitution, which leads to a more rigid conformation of the IN_A_ active site, confers higher enzyme activity but reduces the ability to adapt its active site to the G118R mutation. Recombinant IN activity reduced by drug resistant mutations usually corresponds to a reduced replicative capacity of the mutant virus; therefore, we can expect the emergence and fixation of drug-resistant variants of HIV-1 FSU-A carrying the primary mutation Q148K and compensatory mutations E138K and/or G140S, while the emergence and fixation of drug-resistant variants of FSU-A with the G118R substitution are unlikely.

## Abbreviations

HIV-1: human immunodeficiency virus type 1
IN: integrase
INA: integrase of HIV-1 subtype A strain FSU-A
INB: integrase of HIV-1 subtype B strain HXB-2
RAL: raltegravir
EVG: elvitegravir
DTG: dolutegravir
IC50: inhibitor concentration causing 50% decrease in enzymatic activity
FC: fold change in IC50 of a mutant protein compared to that of wild-type integrase
wt: wild-type integrase
PAAG: polyacrylamide gel
DTT: dithiothreitol
EDTA: ethylenediaminetetraacetic acid
TBE: tris-borate-EDTA buffer
